# A Biomimetic Miniaturized Microphone Array for Sound Direction Finding Applications Based on a Phase-Enhanced Electrical Coupling Network

**DOI:** 10.3390/s19163469

**Published:** 2019-08-08

**Authors:** Chien-Chang Huang, Chien-Hao Liu

**Affiliations:** Department of Mechanical Engineering, National Taiwan University, Taipei 10617, Taiwan

**Keywords:** biomimetic miniaturized microphone array, sound direction finding, sonar, coupling network, Ormia Ochracea

## Abstract

In this research, we proposed a miniaturized two-element sensor array inspired by Ormia Ochracea for sound direction finding applications. In contrast to the convectional approach of using mechanical coupling structures for enlarging the intensity differences, we exploited an electrical coupling network circuit composed of lumped elements to enhance the phase differences and extract the optimized output power for good signal-to-noise ratio. The separation distance between two sensors could be reduced from 0.5 wavelength to 0.1 wavelength 3.43 mm at the operation frequency of 10 kHz) for determining the angle of arrivals. The main advantages of the proposed device include low power losses, flexible designs, and wide operation bandwidths. A prototype was designed, fabricated, and experiments examined within a sound anechoic chamber. It was demonstrated that the proposed device had a phase enhancement of 110${}^{{}^{\circ}}$ at the incident angle of 90${}^{{}^{\circ}}$ and the normalized power level of −2.16 dB at both output ports. The received power levels of our device were 3 dB higher than those of the transformer-type direction-finding system. In addition, our proposed device could operate in the frequency range from 8 kHz to 12 kHz with a tunable capacitor. The research results are expected to be beneficial for the compact sonar or radar systems.

## 1. Introduction

Recently, miniaturizatioxns of sensor or antenna arrays have attracted lots of attention in the areas of military and modern communications for providing indoor/outdoor localizations [1,2], beam scanning, searching, and direction finding [3]. The wide applications include underwater sonar [4,5], radar system [6], seismic monitoring [7], and smart antennas [8]. Direction finding techniques have long been used to determine the angel of arrivals (AOA) of incident waves via the received time differences or intensity differences of each element of the arrays. In general, the distances between each element of an array should be at least half a wavelength for obtaining a distinguishable phase difference or time difference in order to determine the AOA [9]. However, for low frequency applications, the sizes of arrays can be very large in the ranges of km such as very high frequency (VHF) antenna arrays or acoustic sonar arrays. Various researchers have investigated miniaturized arrays in order to reduce the size of array and maintain accurate determinations of AOA. Miniaturized arrays for AOA determinations become an important topic in the acoustic and radio frequency (RF) fields.

Most mammals with hearing capabilities can locate sound sources based on the interaural time differences (ITDs) or interaural intensity differences (IIDs) received by two acoustic cues due to large-size heads [10]. For tiny insects such as Ormia Ochracea, Miles et al. found that Ormia Ochracea with the size of 1 cm could distinguish the AOA of the incident sounds with the wavelength of 7 cm attributed to the coupling mechanisms within their front heads [11,12,13]. In fact, the coupling structures of Ormia Ochracea were composed of spring, mass, and damper which could amplify the ITDs or IIDs between two ears for AOA determinations. Therefore, Ormia Ochracea inspired coupling mechanisms are beneficial for reducing the receiving apertures of microphone or sensors arrays. Various applications have been developed such as Microelectromechanical Systems (MEMS) directional microphones [14,15,16,17], MEMS direction finding sensors [18,19,20,21,22], ultrasonic transducers [23], optical-based directional microphones [24,25], optical sensors [26], and micromachined diaphragms [27,28,29]. Based on the mechanical-coupling induced two vibration modes such as rocking and bending modes, the differences of the time and intensities of two output membranes can be utilized to determine the AOA where the bending mode happens when the two membranes move in the same direction and the rocking mode happens when they move in opposite directions. One disadvantage of mechanical coupling structures is that they often require complicated manufacturing processes and can not be easily adjusted for operating in different frequencies [30].

Based on the analogy between the mechanical and electrical engineering, the mechanical coupled-ear structure can be substituted with an equivalent electrical coupling circuit. Xu et al. proposed an Ormia Ochracea inspired miniaturized microphone arrays with an electrical coupling network composed of transformers, inductors, and capacitors for sound source localizations [31,32]. The equivalent coupling circuit included two excitation modes such as the common and differential modes that were similar to the two aforementioned resonant modes of the mechanical coupled-ear. Via the common and differential modes, the received intensities of the outputs were amplified for detections of incident angles of sounds where the two microphones were separated with a small distance of 7 mm (i.e., 0.4 wavelength) [31]. However, transformer-based coupling circuits might cause additional losses within the circuit and reduce the available power level at the outputs. Zhang et al. developed an electrical coupling filter with two inputs and two outputs for miniaturized microphone arrays [30]. In addition to sound localizations, Behdad et al. applied electrical coupling circuits to miniaturizations of antenna arrays for direction finding applications [33,34,35,36,37,38,39]. Instead of enlarging the intensity differences, they focused on the phase enhancements between two antennas for determining the AOA of incident electromagnetic waves. Akcakaya et al. presented a biologically inspired miniaturized antenna array based on the multi-input-multi-output filter and Cramer–Rao lower bound technique [40].

In this research, we proposed a miniaturized two-element sensor array composed of two microphones acting as the receiving sensors and a pure lumped-element coupling network circuit for acoustic directional finding applications. The two microphones were separated from each other with a distance of 0.1 wavelength 3.43 mm) corresponding to the operation frequency of 10 kHz. The electric coupling circuit was a four port coupling network with two inputs and two outputs where the two inputs were connected to the two microphones. There was a trade-off between the phase differences and the extracted power at the two output ports. By appropriately matching the common and differential modes of the excitations, the coupling circuit could extract the maximum received power at the two output ports. On the contrary, the output amplitude of the differential mode should be maximized to obtain a large phase enhancement. Compared to conventional sensor arrays with the separation distance of at least of 0.5 wavelength, our proposed coupling network could sacrifice a small amount of power to enhance the output phase difference for miniaturization. Therefore, the coupling network circuit was optimized to have a relative large phase enhancement and extract the maximum received power at two output ports. The advantages of the proposed miniaturized sensor array include low power losses, flexible designs, easy fabrications, and wide operation bandwidths.

This paper is organized as follows. In the next section, we present the miniaturized directional finding system and the design procedures of the electrical coupling network which are optimized to have a large phase enhancement and extract the maximum received power. A prototype is designed, fabricated, and experimentally examined within an anechoic chamber for characterizing the system. Subsequently, our proposed device is compared with a transformer-based acoustic direction finding system. Then, a tunable lumped element is applied in our coupling network circuit to increase the operation frequency ranges. Important results are summarized at the end of the paper.

## 2. Biomimetic Miniaturized Microphone Array

**Figure 1:**
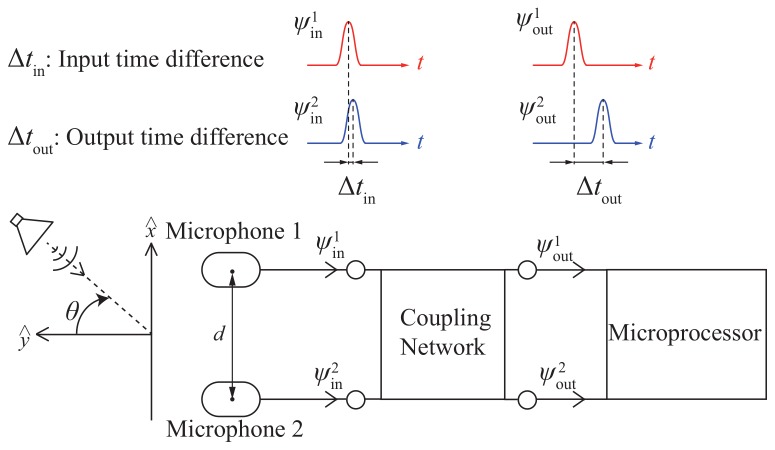
Block diagram of the two-element microphone array where the two microphone is separated with a small distance, *d*. Based on the electrical coupling network circuit, the input time difference received by the two microphones are amplified at the outputs for determining the incident angles of sounds.

Figure 1 shows the block diagram of the biomimetic miniaturized microphone array composed of two commercial available microphones and an electrical coupling network circuit. The two microphones have omnidirectional patterns and are separated with a distance of *d* for receiving sounds. When illuminated by a plane sound wave with an incident angle of θ, there is a time difference, $\Delta t_{in}$, between the received sound waves, $\psi_{in}^{1}$ and $\psi_{in}^{2}$, obtained from the two microphones due to the separation distance. Where θ is measured from the boresight direction and θ=0 indicates a normal incident sound wave. As the separation distance decreases, the time difference and corresponding phase difference, $\phi_{out}$ (θ) = *∠*$\psi_{out}^{2}-∠$$\psi_{out}^{1}$, become small. Then, the received sound signals are delivered to the electrical coupling network circuit for phase enhancement. Via an optimized matching of the common and differential modes, the input phase difference received by the two microphones are amplified at the outputs of the coupling network circuit. The output sound signals, $\psi_{out}^{1}$ and $\psi_{out}^{2}$, with a large time difference, $\Delta t_{out}$, and the corresponding phase difference, $\phi_{out}$ (θ) = *∠*$\psi_{out}^{2}-∠$$\psi_{out}^{1}$, are transferred to the microprocessor for determining the incident angles of sounds.

### 2.1. Designs of Coupling Network Circuit

**Figure 2:**
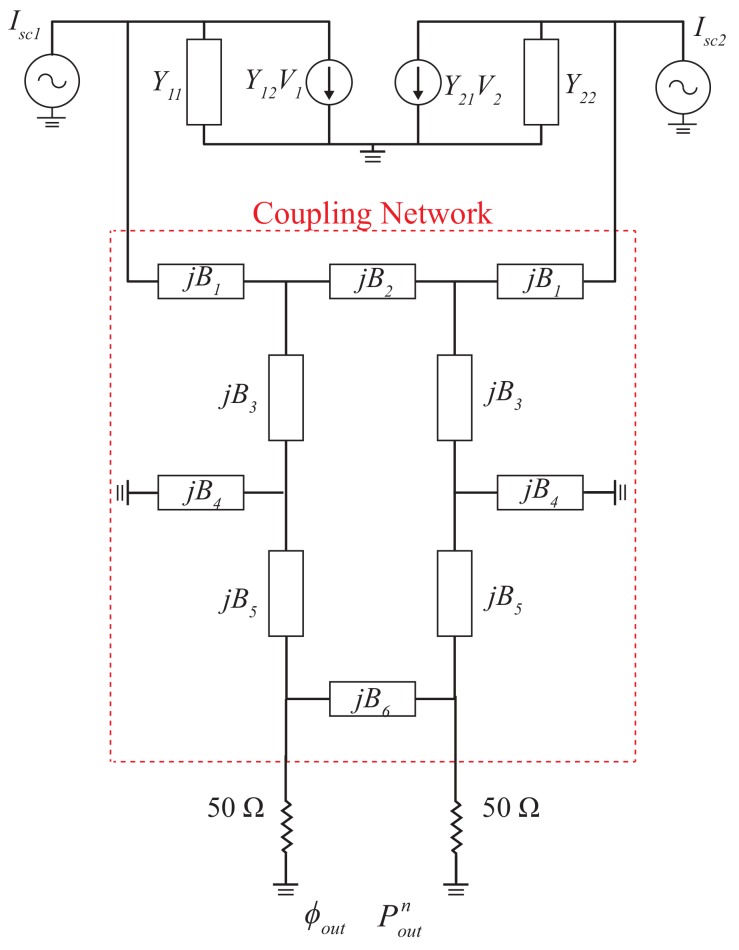
The equivalent circuit model of the miniaturized microphone array where each microphone is modeled as a Norton equivalent current source. The mutual coupling between two adjacent microphones is modeled as a voltage dependent current source and the electrical coupling network is composed of 10 lumped elements.

Figure 2 shows the equivalent circuit model of the proposed miniaturized microphone array composed of two microphones and an electrical coupling circuit shown in Figure 1. If two microphones are placed closely to each other, the mutual coupling effect plays an important role in the received sound signals. This mutual coupling can be utilized to enhance the output phase difference of the microphone array via a coupling network circuit described later.

In general, the frequency response of a single microphone is characterized by its impedance. For taking into account the mutual coupling effect, we introduce the well-known networking concept of the microwave engineering for characterizing the two-microphone array with an admittance matrix expressed below [35]:

(1) $$Y_{11}=G_{11}+jB_{11},$$

(2) $$Y_{12}=G_{12}+jB_{12},$$

where an admittance is defined as the inverse of the impedance. $G_{11}$($B_{11}$) is the real (imaginary) part of the admittance of each microphone and $G_{12}$($B_{12}$) is the real (imaginary) part of the mutual admittance between two microphones. The two outputs of the microphone array are connected to the electrical coupling network as shown in Figure 2.

The electrical coupling network circuit is a four-port network with two inputs connected to the microphone array and two outputs connected to the loads or the microprocessor. Based on the microwave engineering theory, the coupling network can be described by the scattering matrix represented as a ${\left[S\right]}_{4\times 4}$ matrix containing ten independent variables. With the assumption of being lossless, reciprocal, and symmetric, the coupling network is simplified with six independent variables, $B_{1}$ to $B_{6}$ which can be optimized to provide a phase enhancement and an adequate power at output ports.

**Figure 3:**
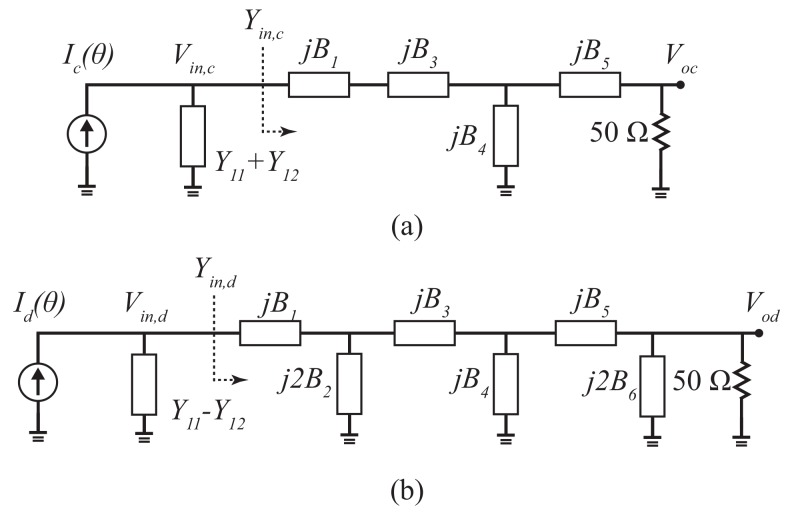
The equivalent circuit model of (**a**) common mode and (**b**) differential mode.

Each microphone is modeled as a Norton equivalent current source and the mutual coupling between two adjacent microphones is modeled as a voltage control current source. The two microphones are assumed to be identical under illuminations of a plane sound wave, the input signals received by individual microphones, $I_{sc1}=e^{j\alpha }$ and $I_{sc2}=e^{-j\alpha }$, have the same amplitude, but a phase difference of 2α due to the separation distance where α=πdsin(θ)/λ. The input signals obtained from the microphone array can be represented as the common mode and differential mode [41] shown in Figure 3 expressed as:

(3) $$I_{c}=\frac{I_{sc1}\left(\theta \right)+I_{sc2}\left(\theta \right)}{2},$$

(4) $$I_{d}=\frac{I_{sc1}\left(\theta \right)-I_{sc2}\left(\theta \right)}{2},$$

where $I_{c}$ ($I_{d}$) is the common (differential) mode short-circuit current. The common and differential mode short-circuit currents are functions of the incident angles. For incident angles close to boresight direction (θ≈0), these short-circuit currents can be further simplified as:

(5) $$I_{c}{|}_{\theta =0}\approx 1,$$

(6) $$I_{d}{|}_{\theta =0}\approx j\alpha .$$

As shown in Figure 3a, the maximum available power obtained by the microphone array can be transferred to the 50 Ohms loads by matching the electrical coupling network with the admittance of the microphone array in the common modes. With the assumption of the lossless circuit, no power is consumed within the coupling circuit and the magnitude of the output common mode voltage has a maximum value of $|V_{oc}^{max}|$.

To characterize the phase enhanced sensitivity of the coupling network, the output phase differences of the miniaturized microphone array consisted of coupling network circuit is compared with that of the regular microphone array without the coupling network where each microphone is separated from each other with the same distance of the miniaturized microphone array. Therefore, the phase enhancement factor is defined as $\eta =s/s_{0}$, where *s* is the slope of the output phase of the coupling network circuit and $s_{0}$ is the slope of the output phase of the regular microphone array without the coupling network circuit. For normal incident sound waves (i.e., boresight direction), *s* and $s_{0}$ can be expressed as:

(7) $$s=\frac{d\phi_{out}}{d\theta }=\left(\frac{d\phi_{out}}{d(2\alpha )}{|}_{\alpha =0}\right)\left(\frac{d(2\alpha )}{d\theta }{|}_{\theta =0}\right),$$

(8) $$s_{0}=\frac{d}{d\theta }\left(2\alpha \right){|}_{\theta =0}=2\pi \frac{d}{\lambda },$$

where the output phase difference $\phi_{out}$ is the substraction of phases between the two output voltage, $V_{o1}$ and $V_{o2}$. Then, the *s* can be expressed as:

(9) $$\begin{array}{cc} \eta & ≜\lim_{2\alpha \to 0}\left[\frac{∠V_{o1}-∠V_{o2}}{2\alpha }\right]\\ & =\lim_{2\alpha \to 0}\left[\frac{1}{2\alpha }∠\frac{V_{oc}+V_{od}}{V_{oc}-V_{od}}\right]\\ & =Re\left[\frac{1}{j\alpha }\frac{V_{od}}{V_{oc}}\right]. \end{array}$$

The phase enhancement factor depends on the output common and differential voltages. Since the output common mode voltage is a constant and the output differential mode voltage is a function of incident angle, the phase enhancement factor can be maximized by enlarging the magnitude of the output differential mode voltage and the phase between the differential mode and common mode should be 90${}^{{}^{\circ}}$. In other words, there is a maximum value of the phase enhancement factor by both matching the common and differential modes. However, there is a trade-off between the maximum phase enhancement and the output power. The goal of this research is to design the coupling network circuit for the microphone array with a large phase enhancement which is not the maximum value and an acceptable output power. To characterize the output power at both 50 ohms loads for different incident angles of sounds, the normalized output power, $P_{out}^{n}$, is defined as:

(10) $$P_{out}^{n}=\frac{P_{out}}{P_{0}},$$

where $P_{out}$ is the output power of each output load of the coupling network circuit. $P_{0}$ is the available power obtained by each element of the microphone array for normal incident angles.

### 2.2. Increase the Operation Frequency Bandwidth

**Figure 4:**
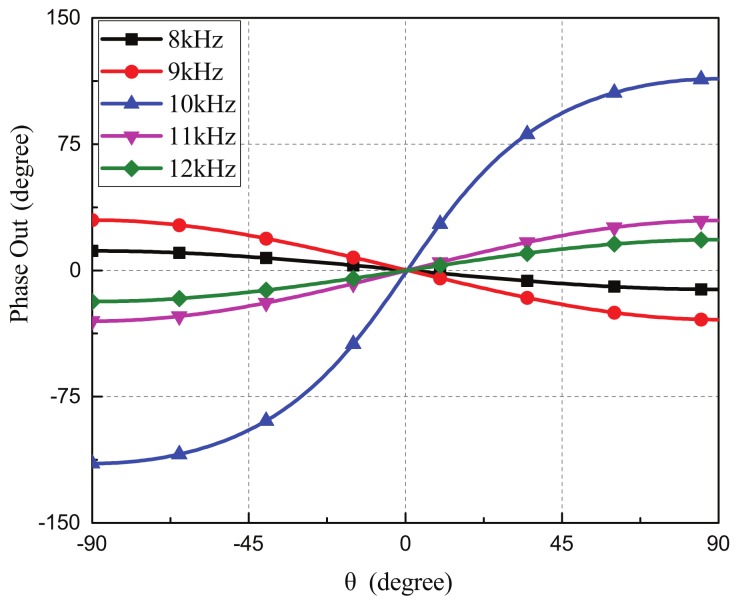
The simulated output phase enhancement of the microphone array with the pure-lumped-element based coupling network operated at different frequencies from 8 to 12 kHz. The coupling network only provided a phase enhancement at the designed operation frequency where the common and differential modes were matched appropriately.

For the aforementioned miniaturized microphone array, the coupling network circuit was designed to have a large output phase enhancement operating at a narrow frequency range by matching the common and differential modes. The narrow operation bandwidth occurred for most mechanical [25,42] and electrical coupling networks [39,43]. This was due to the fact that, if the operation frequency deviated from the desired frequency, the output phase difference was not enhanced due to the mismatches of the common and differential modes. Figure 4 shows the simulated output phase differences of the our device operated at different frequencies from 8 to 12 kHz. The electrical coupling network was a narrow band circuit and provided a phase enhancement at the designed frequency of 10 kHz. For other operation frequencies, no phase enhancements were observed. Similar to other bio-inspired direction finding devices, the miniaturized microphone array with a narrow band coupling network can not be used for incident sounds with wide bandwidth.

**Figure 5:**
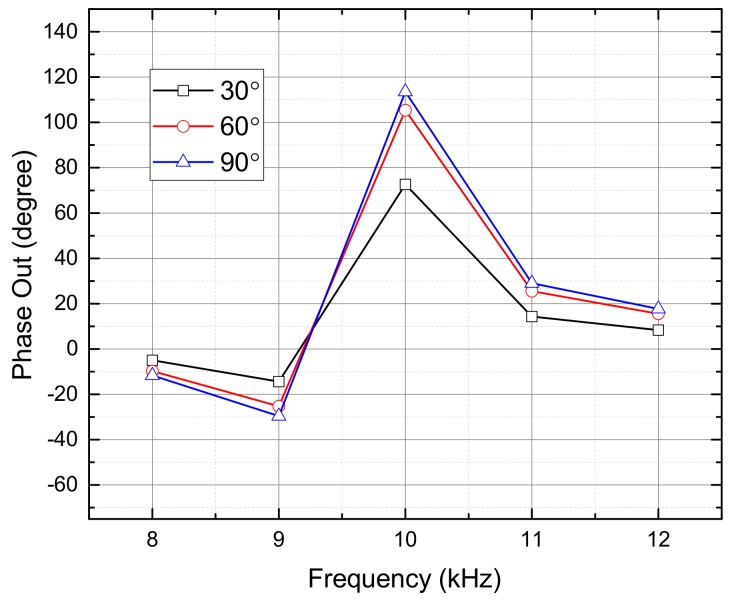
The simulated output phase differences versus different frequencies for the incident angles of 30${}^{{}^{\circ}}$, 60${}^{{}^{\circ}}$, and 90${}^{{}^{\circ}}$.

Figure 5 shows the simulated output phase differences versus different frequencies for the incident angles of 30${}^{{}^{\circ}}$, 60${}^{{}^{\circ}}$, and 90${}^{{}^{\circ}}$. As can be observed, the output phase differences was enhanced significantly near 10 kHz and relatively small when deviated from 10 kHz, indicating that the proposed circuit network had a narrow operation bandwidth.

**Figure 6:**
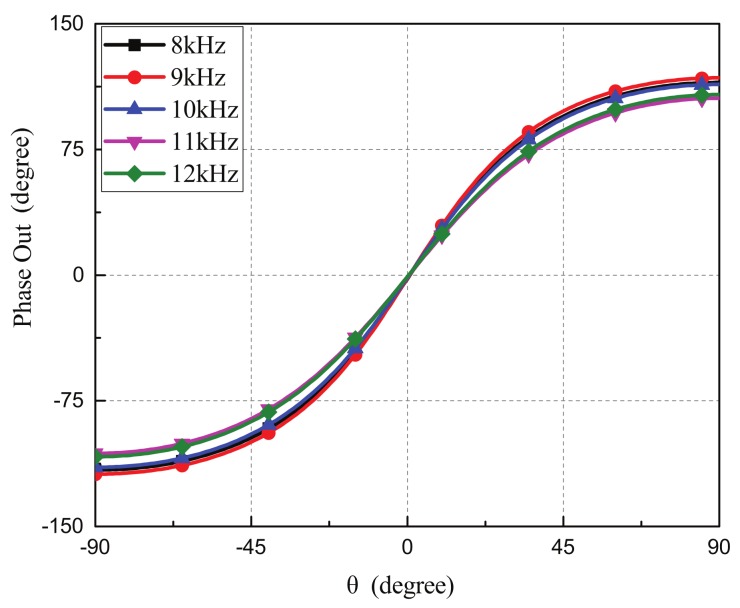
The simulated output phase enhancement of the microphone array with an electronic tunable coupling network circuit that could can provide a wide operation frequency range from 8 to 12 kHz.

For increasing the operation bandwidth of the miniaturized microphone array, we proposed an electronic tunable coupling network circuit by substituting the fixed-value lumped elements with electronic tunable lumped elements. The idea was to match the common and differential modes for output phase enhancements at different frequencies via adjusting the values of the lumped elements. Since the output phase enhancement was sensitive to the Y parameter of $B_{6}$, we chose the $B_{6}$ element as the tunable element for the wideband design of the coupling network circuit. The goal was to tune the value of the element to have nearly the same output phase enhancements at different frequencies where the values of $B_{6}$ at different operation frequencies were listed in Table 1. Figure 6 shows the simulated output phase enhancement of the microphone array with an electronic tunable coupling network circuit which can provide a wide operation frequency range from 8 to 12 kHz. Therefore, the bio-inspired microphone array with a tunable coupling network circuit can be exploited for wideband direction finding applications. Note that the separation distance between two microphones remained the same value for different operation frequencies since both microphones were mounted on the PCB board with a fixed distance. For the tunable frequency range from 8 kHz to 12 kHz, the fixed distance corresponded to 0.08 wavelength to 0.12 wavelength indicating that the microphone array had a miniaturized dimension of approximate 0.1 wavelength. For broad band applications, the separation distance could be varied to maintained 0.1 wavelength to achieve miniaturized microphone arrays.

## 3. Fabrications and Measurements

### 3.1. Microphones

**Figure 7:**
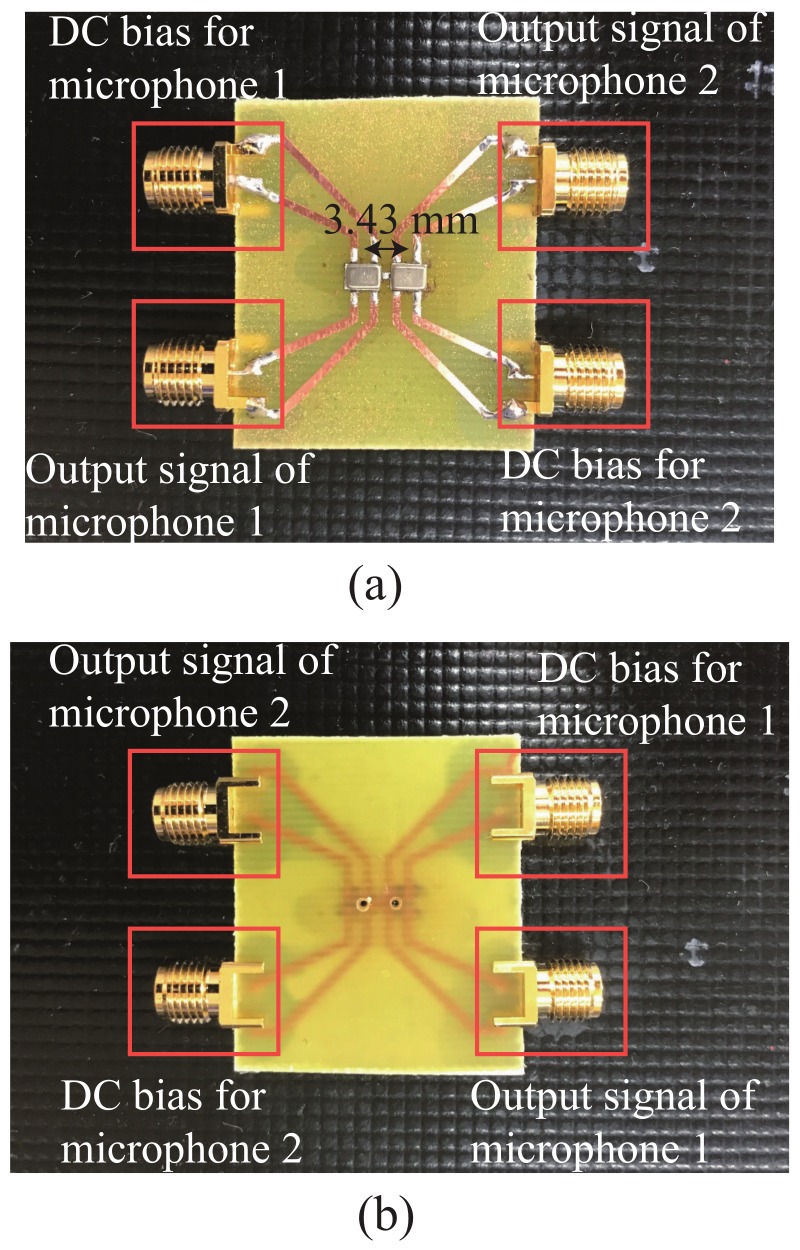
The photographs of (**a**) the front side and (**b**) the back side of the microphone array.

The miniaturized microphone array was designed to operate at the frequency of 10 kHz for sound direction finding applications. We exploited two commercial-available MEMS analog microphones (model: MMA204 from Merry Inc., Taiwan). The characteristics of MMA204 microphones included the sensitivity of −38 dB (0 dB = 1 V/Pa), frequency response from 50 Hz to 20 kHz, signal-to-noise ratio of 65 dB, required supply voltage from 1.5 V to 3.6 V, and size of 3.35 mm × 2.5 mm ×0.98 mm. The two microphones were surface mounted on a FR-4 PCB substrate with a separation distance of 3.43 mm (i.e., 0.1$\lambda_{0}$) where $\lambda_{0}$ is the wavelength of the incident sound wave. The FR-4 PCB substrate used throughout this research had the dielectric constant of $\epsilon_{r}$ = 4.4 and a thickness of 1.6 mm. Figure 7a,b show the front and back sides of the two microphones, respectively. direct current (DC) bias voltages of 3.3 V were provided to the two microphones. Since the sound detecting components were on the bottom of the microphones, two holes were drilled for receiving the incident sounds on the back side of the PCB board. A calibrated low-frequency vector network analyzer (model: E5061B from Keysight Tech., SR, CA, USA) was utilized to measure the impedance matrix ([Z]) of the fabricated microphone array. The measured Y matrix (i.e., admittance matrix) converted from the scattering matrix was shown in Table 2. $G_{11}$ and $B_{11}$ were the real and imaginary parts of the admittance component, $Y_{11}$. $G_{12}$ and $B_{12}$ were the real and imaginary parts of the admittance component, $Y_{12}$. These values were exploited for designing the coupling network circuit.

### 3.2. Coupling Network Circuit

**Figure 8:**
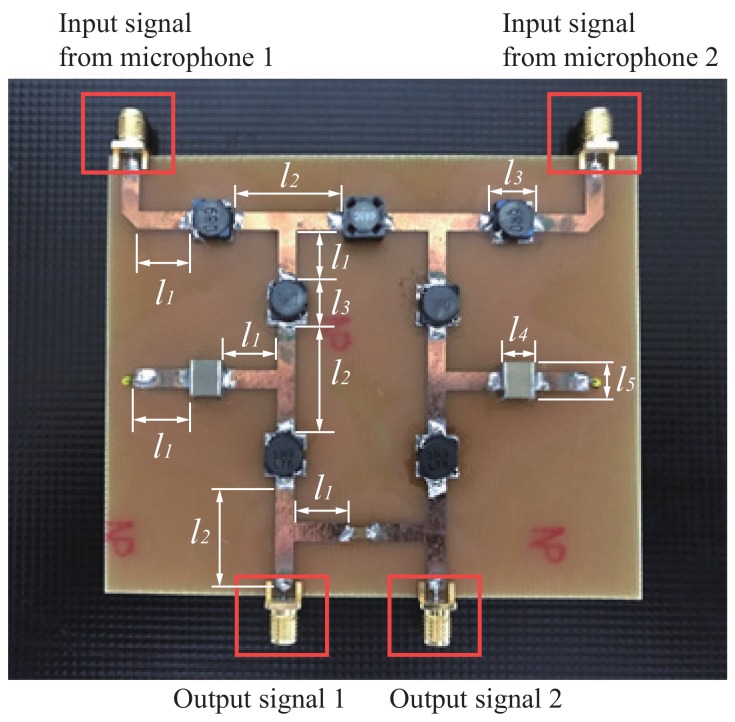
The coupling network circuit implemented with 10 lumped elements and transmission lines on the FR4 printed circuit board (PCB) substrate.

Based on Equation (9), the phase enhancement factor was calculated and chose as η = 10 for the coupling network circuit. The two inputs of the coupling network circuit were connected to the two outputs of the microphone arrays shown in Figure 7. The coupling network circuit was composed of six independent Y-parameters including $B_{1}$ to $B_{6}$. The goal was to obtain a phase difference of 100 degrees between two output signals of the coupling network circuit and extract maximum output power for direction finding applications. Via an optimization of circuit simulations, the Y parameters of $B_{1}$ to $B_{6}$ were obtained and listed in Table 3. Then, the ideal Y parameters were transformed into practical capacitances and inductances operating at the frequency of 10 kHz. The coupling network circuit was implemented with 10 surface mounted inductors and capacitors fabricated on the FR4 PCB substrate connected by microstrip lines. Figure 8 shows the photograph of the coupling network circuit composed of surface mounted lumped element implemented via the FR-4 PCB substrate and microstrip lines. The geometric dimensions of the transmission lines were provided in Table 4.

### 3.3. Field Test Examinations

**Figure 9:**
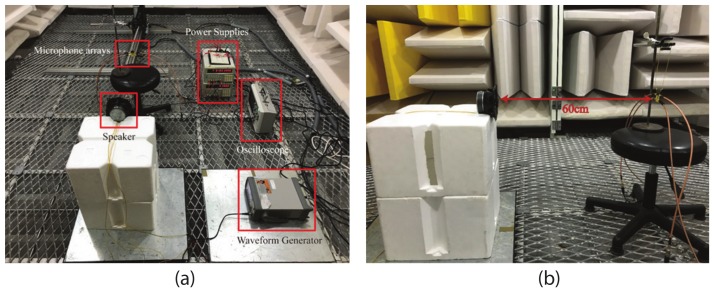
(**a**) the experiment setup for the field tests of the microphone array; (**b**) side view indicating the distance between the speaker and the microphone array.

**Figure 10:**
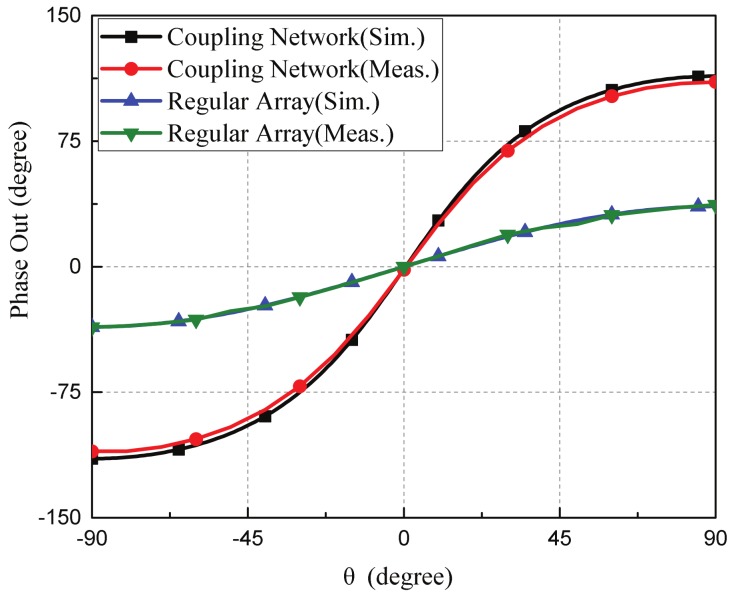
The measured and simulated output phase differences of the proposed miniaturized microphone array with the coupling network circuit and the regular microphone array without the coupling network.

The field tests of the fabricated miniaturized microphone array were conducted in the sound anechoic chamber at the acoustic lab of Science and Ocean Engineering Department, National Taiwan University. Figure 9a shows the experimental setup. The speaker and the microphone array were separated with a distance of 60 cm (i.e., 18λ) for incident plane waves shown in Figure 9b. The waveform generator (model: Agilent, SCC, CA, USA 33500B Series) was exploited to excite the commercial available speak (Pioneer TS-D1602R, Torrance, CA, USA) for generating continuous sound waves with the sound pressure level of 80 dB. Two DC power supplies (model: GWinstek PPE-3323 DC) were utilized for the MEMS microphones with 3.3 V and he amplifier circuit with ±15 V. The oscilloscope (model: Keysight DSOX2012A) with two synchronized channels was used to extract the phase differences between two received signals. Figure 10 shows the simulated and measured phase differences between the two outputs of the coupling network circuit and a regular microphone without the coupling network for an incident angle of sound wave from –90${}^{{}^{\circ}}$ to 90${}^{{}^{\circ}}$. The incident angle was varied and measured every 10${}^{{}^{\circ}}$. Since the separation distance between the two microphones of the regular two-element microphone array was smaller than half wavelength, the simulated and measured output phase differences were small and could not be used for determining the AOA. For our device with a coupling network circuit, the simulated output phase differences were 113${}^{{}^{\circ}}$ for the incident angle of 90${}^{{}^{\circ}}$ and −114${}^{{}^{\circ}}$ for the incident angle of −90${}^{{}^{\circ}}$. The measured output phase differences of our devices were 110.12${}^{{}^{\circ}}$ for the incident angle of 90${}^{{}^{\circ}}$ and −110.34${}^{{}^{\circ}}$ for incident angle of −90${}^{{}^{\circ}}$. The simulation results matched with the measurement results. There was a small output phase difference of −1.78${}^{{}^{\circ}}$ for normal incident ($\theta =0^{{}^{\circ}}$) due to the fabrication tolerances and imperfect symmetry.

**Figure 11:**
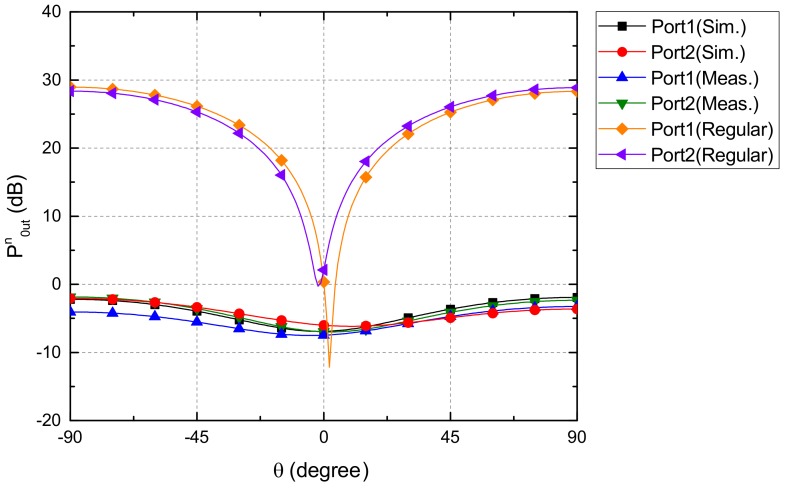
The measured and simulated normalized output power at each output port of the coupling network circuit of the miniaturized microphone array. The simulated normalized output power of the miniaturized microphone array was provided for comparisons.

The simulated and measured normalized output power of each output port of our device for different incident angles was shown in Figure 11. The measurement and simulation results had a similar trend with some deviations. For output port 1, the maximum measured normalized output power was −2.16 dB for the incident angle of 90${}^{{}^{\circ}}$ which was slightly smaller than the simulated result of −1.92 dB. The minimum measured normalized output power was −6.75 dB for the normal incident angle ($\theta =0^{{}^{\circ}}$) which was higher than the simulated value. For output port 2, the maximum measured normalized power was −2.74 dB for the incident angle of 90${}^{{}^{\circ}}$ similar to the measured results of output port 1. The minimum measured normalized power was −7.13 dB for the normal incident sound ($\theta =0^{{}^{\circ}}$) which was lower than the simulated value. The discrepancies between the simulated and measured results might be due to the fabrication tolerances, imperfect symmetry, and values deviations of the off-shelf lumped elements. In addition, the simulated normalized output power of each output port of the microphone array without the coupling network circuit was provided for comparisons. It could be observed that our proposed device scarified partial output power and maintained sufficient output power for phase enhancements.

### 3.4. Compared with Transformer-Type Microphone Arrays

In general, Ormia Ochracea-inspired mechanical coupling networks are composed of springs, masses, and dampers where dampers can consume some input acoustic energy and reduce the maximum available input energy obtained from the sensors or microphones. Analogously to the mechanical coupling network, the equivalent electrical coupling network circuits are consisted of lossy transformers and lumped-elements for mimicking the hearing mechanisms where transformers can transform the current outputs to voltage outputs of the coupling circuits [31,32,33]. Although the lossy electrical coupling network can provide a large output phase difference for direction findings applications, it can not extract the full available energy obtained from the sensors or antennas to the output port. In other words, some energy is consumed within the lossy coupling network circuit.

**Figure 12:**
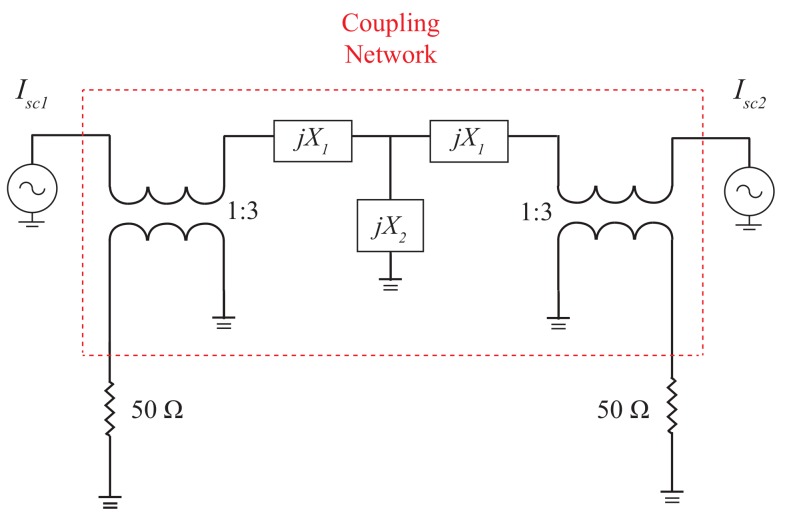
The equivalent circuit model of the transformer-based microphone array where the coupling network was composed of lossy transformers and lumped elements.

In this section, we compared the pure-lumped-element based coupling network and the transformer-based coupling network in terms of the output phase differences and the normalized output power for developing miniaturized microphone array. Figure 12 shows the equivalent circuit model of the transformer-based microphone array where the coupling network was composed of transformers with 1:3 turn ratio and lumped elements. The two microphones were separated with a distance of 0.1 wavelength and the two input sound sources obtained from the two microphones were $I_{sc1}=e^{\left(j\alpha \right)}$ and $I_{sc1}=e^{\left(-j\alpha \right)}$. For comparing the output power of the lossless and lossy coupling network, the transformer-based coupling circuit was designed to have the same output phase differences as our device. The electrical parameters of the transformer-based coupling network circuit were $X_{1}$ with the value of 21 S and $X_{2}$ with the value of 20 S.

**Figure 13:**
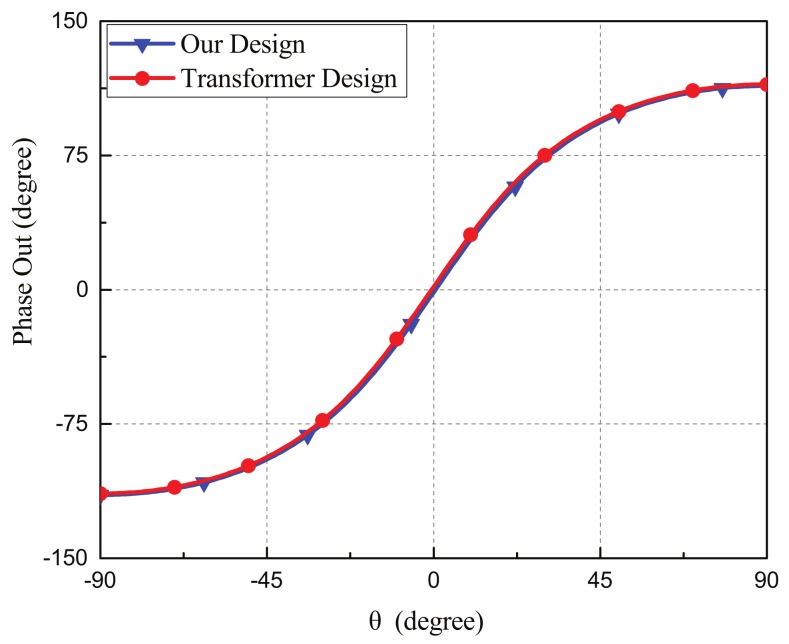
The simulated phase differences of our proposed work and the transformer-based microphone array.

The simulated output phase differences for different incident angles of our device and the transformer-based microphone array were shown in Figure 13. Both of them had similar output phase differences verse the incident angles of sounds. For our device, the output phase differences were 113.78${}^{{}^{\circ}}$ for incident angle of 90${}^{{}^{\circ}}$ and −114.72${}^{{}^{\circ}}$ for the incident angle of −90${}^{{}^{\circ}}$. For the transformer-based coupling network, the output phase difference were 113.79${}^{{}^{\circ}}$ for the incident angle of 90${}^{{}^{\circ}}$ and −114.74${}^{{}^{\circ}}$ for the incident angle of −90${}^{{}^{\circ}}$.

**Figure 14:**
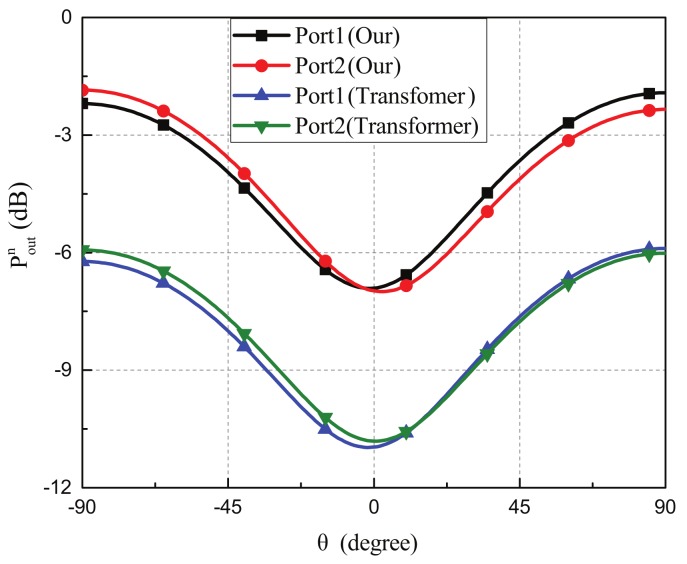
Comparisons of the simulated normalized output power at two output ports between our device and the transformer-based microphone array. The output power of the transformer type microphone array was approximately 4 dB below than that of our device due to power loss of the transformers.

Figure 14 shows the comparisons of the simulated normalized output power between our device and the transformer-based microphone array. For output port 1, the maximum normalized power of our device and the transformer-based microphone array were −1.92 dB and −5.89 dB, respectively, for the incident angle of 90${}^{{}^{\circ}}$. For output ports 2, the maximum normalized power of our device and the transformer-based microphone array were −1.85 dB and −5.82 dB, respectively, for the incident angle of −90${}^{{}^{\circ}}$. The output power of the transformer type microphone array was approximately 4 dB below that of our device due to power loss of the transformers.

## 4. Conclusions

In this research, we presented a biomimetic miniaturized two-element microphone array with an electrical coupling network circuit for enhancing the output phase differences for sound direction finding applications. Two microphones were separated with a small distance of 0.1 wavelength and the small phase differences were amplified by matching the common and differential modes. A prototype was designed, fabricated, and experimentally examined within an acoustic anechoic chamber. The experimental results demonstrated a large phase enhancement and acceptable normalized output power. The coupling network can be improved by including tunable elements for wideband applications. The proposed miniaturized microphone array can be expanded to more than two elements for more accurate determinations of angle of arrivals. It was demonstrated that the proposed coupling network circuit could enhance the output phase differences of microphone arrays. The prototype based on RF4 PCB substrate was a proof of concept which could be further miniaturized with an ASIC chip.

## Figures and Tables

**Table 1:** The tuning values of $B_{6}$ in the frequency range from 8 to 12 kHz.

Freq. (kHz)	8	9	10	11	12
$B_{6}$ (μF)	4.4	3.4	2.7	2.2	1.8

**Table 2:** The measured Y matrix of the fabricated two-element microphone array.

DMMA204 Microphone Array	Unit: Millisiemens (mS)
$G_{11}=Re\left(Y_{11}\right)$	−1.27
$G_{12}=Re\left(Y_{12}\right)$	18.7
$B_{11}=Im\left(Y_{11}\right)$	−0.132
$B_{12}=Im\left(Y_{12}\right)$	0.328

**Table 3:** Values of the six Y-parameters of the coupling network circuit exploited in circuit simulations (unit: S).

$B_{1}$	$B_{2}$	$B_{3}$	$B_{4}$	$B_{5}$	$B_{6}$
2.65	0.23	5.31	−0.08	4.08	−0.17

**Table 4:** Geometric dimensions of the microstrip lines (unit: mm).

$l_{1}$	$l_{2}$	$l_{3}$	$l_{4}$	$l_{5}$
10	20	7.2	5.5	6.3
